# Expectations for the Dual Therapy with Vonoprazan and Amoxicillin for the Eradication of *H. pylori*

**DOI:** 10.3390/jcm12093110

**Published:** 2023-04-25

**Authors:** Takahisa Furuta, Mihoko Yamade, Tomohiro Higuchi, Satoru Takahashi, Natsuki Ishida, Shinya Tani, Satoshi Tamura, Moriya Iwaizumi, Yasushi Hamaya, Satoshi Osawa, Ken Sugimoto

**Affiliations:** 1 Furuta Clinic for Internal Medicine, 1963-15 Mitsuke, Iwata, Shizuoka 438-0086, Japan; 2 First Department of Medicine, Hamamatsu University School of Medicine, 1-20-1 Handayama, Higashi-ku, Hamamatsu 431-3192, Japan; 3 Clinical Laboratories, Hamamatsu University School of Medicine, 1-20-1 Handayama, Higashi-ku, Hamamatsu 431-3192, Japan; 4 Endoscopic and Photodynamic Medicine, Hamamatsu University School of Medicine, 1-20-1 Handayama, Higashi-ku, Hamamatsu 431-3192, Japan

**Keywords:** *H. pylori*, dual therapy, vonoprazan (VPZ), amoxicillin (AMOX), clarithromycin (CAM)

## Abstract

Vonoprazan (VPZ) inhibits gastric acid secretion more potently than proton pump inhibitors. Recently, attention has been focused on the dual therapy with VPZ and amoxicillin (AMOX) for the eradication of *H. pylori.* The dual VPZ/AMOX therapy attains the sufficient eradication rate with lowering the risk of adverse events in comparison with the triple therapy and quadruple therapy. Therefore, the dual VPZ/AMOX therapy is considered a useful eradication regimen for *H. pylori* infection.

## 1. Introduction

One of the first-line eradication regimens for *H. pylori* has long been a triple therapy with proton pump inhibitor (PPI), amoxicillin (AMOX), and clarithromycin (CAM). However, with the recent increase in CAM-resistant strains of *H. pylori*, the eradication rates of PPI/AMOX/CAM therapy have been declining [1], which is a global problem. The reported incidences of CAM-resistance strains of *H. pylori* are approximately 25–32% [2,3,4]. In addition, since the effects of PPIs are influenced by the genetic polymorphism of CYP2C19, which is the main metabolic enzyme of PPIs, it has been pointed out that the eradication rates in the CYP2C19 extensive metabolizers are lower in comparison with other metabolizers [5,6]. In order to recover the eradication rates of the first-line eradication therapy, bismuth or non-bismuth quadruple therapies are being used, which are recommended especially in areas with high CAM-resistance rates [7]. Although the eradication rates of quadruple therapy are high, multiple drugs must be taken and the frequency of side effects is high.

There have been some reports of dual therapy with PPIs and AMOX as listed in Table 1 [5,8,9,10,11,12,13,14,15,16,17,18,19,20,21,22,23,24,25]. Originally, eradication therapy for *H. pylori* started with classical triple therapy followed by the dual therapy. However, the eradication rates of the dual therapy have varied as shown in Table 1. After the triple therapy for 1 week was developed [14], the dual therapy with PPI and AMOX was no longer the main therapy for *H. pylori* infection.

Vonoprazan (VPZ) has been clinically available since 2015. VPZ inhibits gastric acid secretion more potently than PPIs [26]. The eradication rate of the triple therapy with VPZ 20 mg bid, CAM 200 mg bid, and AMOX 750 mg bid was reported 92.6%, which was significantly higher than that of the triple therapy with lansoprazole 30 mg and the same doses of CAM and AMOX [27]. Interestingly, in this report, the eradication rate by the triple therapy with VPZ, CAM, and AMOX in patients infected with CAM-resistant strains of *H. pylori* was 82.0%, suggesting that the dual therapy with VPZ and AMOX for 1 week can attain the eradication rate higher than 80.0%. Then, the attention has recently been focused on the dual therapy with VPZ and AMOX.

## 2. Gastric Acid Secretion Required for the Dual Therapy with Amoxicillin

AMOX exerts its antibacterial action by binding to penicillin-binding protein (PBP) and inhibiting the synthesis of bacterial cell wall. According to a report examining the relationship between PBP expression in *H. pylori* and pH, *H. pylori* does not proliferate and PBP expression is low at around pH 3.0 [28]. However, at pH 7.4, *H. pylori* proliferates vigorously and the expression of PBP also increases, indicating that the number of targets of AMOX increases, and it is thought that AMOX becomes more effective. In addition, inhibition of gastric acid secretion leads to stabilization of antibacterial drugs in the stomach and increases their concentration in gastric juice [29], which greatly contributes to the success of eradication.

**Figure 1:**
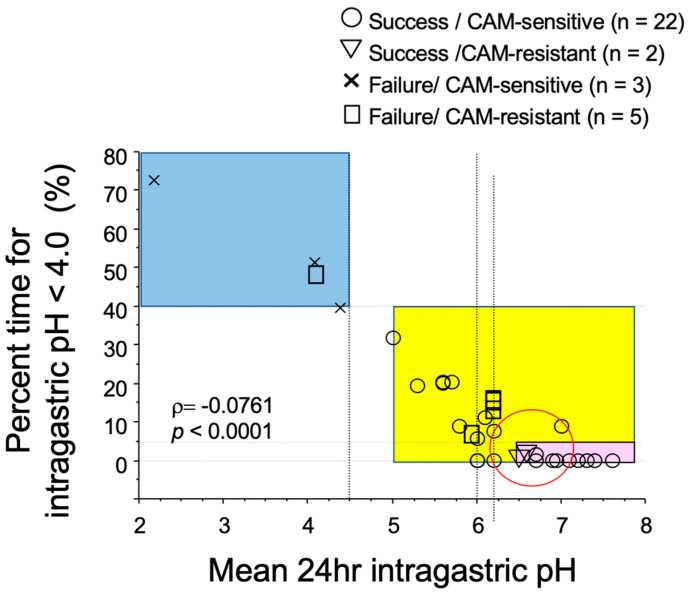
Relationship of success or failure of eradication of *H. pylori* by the triple therapy with PPI, amoxicillin, and clarithromycin (CAM) with CAM-resistance, 24 h intragastric pH, and percent time for intragastric pH < 4.0. When the mean intragastric pH is less than 4.5 and the percent time for intragastric pH < 4.0 was longer than 40% (blue area), eradication of *H. pylori* fails in patients infected with not only CAM-resistant strains (□) but also CAM-sensitive strains (×) of *H. pylori*. When 24 h intragastric pH is higher than 5.0 (yellow area), eradication of *H. pylori* succeeds for CAM-sensitive strains (○) of *H. pylori*. When the mean intragastric pH is no less than 6.5 and the percent time for intragastric pH < 4.0 is less than 5% (pink area), eradication of *H. pylori* succeeds in patients infected with CAM-resistant strains (∇) as well as CAM-sensitive strains (○) of *H. pylori*. Modified [30].

There is a report examining the relationship among the success or failure of eradication by triple therapy, CAM-resistance of *H. pylori* and intragastric pH [30]. When the mean 24-h intragastric pH is less than 4.5 and when the percent time for intragastric pH < 4.0 is longer than 40%, eradication will fail even if *H. pylori* strain is sensitive to CAM (Figure 1 blue area). In contrast, when the mean 24-h intragastric pH exceeds 5, CAM-susceptible bacteria can be successfully eradicated (Figure 1 yellow area). Interestingly, if the mean 24-h intragastric pH in the stomach is higher than 6.5 and the percent time for intragastric pH < 4.0 is 5% or less (pink part in Figure 1), in other words, if the pH 4 holding time ratio (pH 4 HTR) is 95% or more and if the mean 24 h intragastric pH is no less than 6.5, CAM-resistant strains can be eradicated. Therefore, it is suggested that eradication of *H. pylori* can be attained by a single antibiotic, such as AMOX, when the intragastric pH is strictly controlled to be neutral.

**Figure 2:**
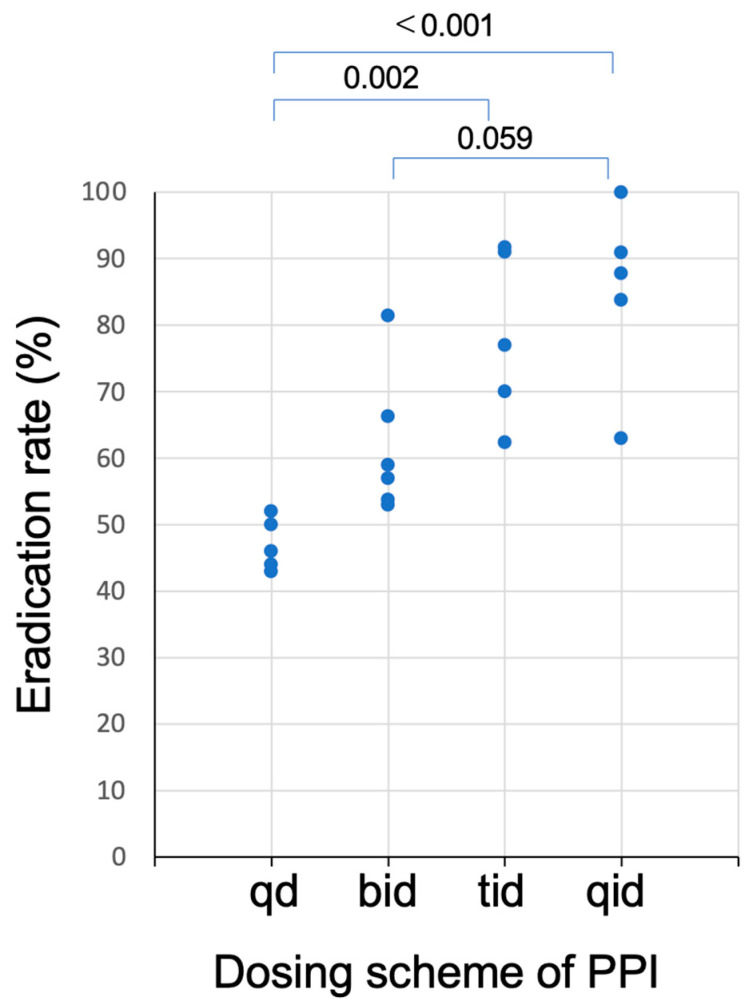
Plots of the eradication rates of studies of dual therapy with PPI and amoxicillin as a function of dosing schemes of PPI listed in Table 1. Eradication rates of regimen with three (tid) or four (qid) times daily dosing of PPI was significantly higher than those with once (qd) or twice (bid) daily dosing.

The relationship between dosing schemes of PPI and eradication rates of the dual therapies with PPI and AMOX listed in Table 1 is plotted in Figure 2. As the dosing frequency of PPI increases, the eradication rates increase. Because the acid inhibitory effect of PPI is enhanced by the divided dosing [31], the eradication rates with dual therapy with PPI and AMOX increase with the grade of acid inhibition.

**Figure 3:**
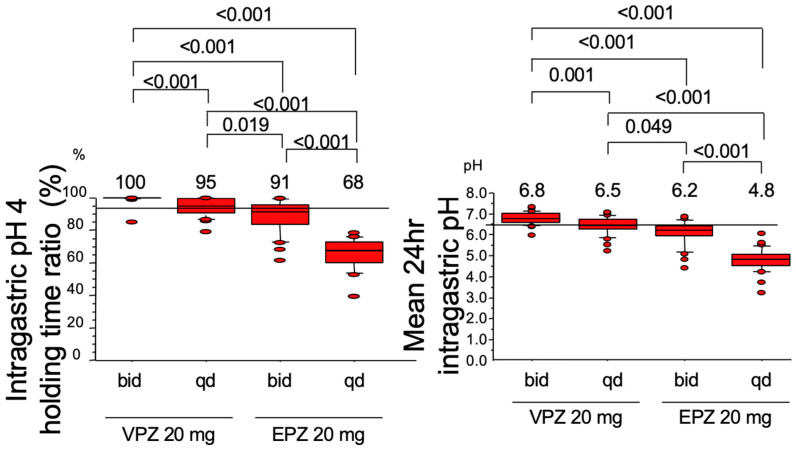
Intragastric pH 4 holding time ratio (**Left** panel) and mean 24 h intragastric pH (**Right** panel) attained by the twice (bid) or once (qd) daily dosing of 20 mg of vonoprazan (VPZ) or esomeprazole (EPZ) on day 7. VPZ 20 mg twice daily can attain both the intragastric pH 4 holding time ratio 100.0% and the mean 24 h intragastric pH 6.8.

Kagami et al. [32] compared the acid inhibitory effects of VPZ and EPZ and found that 95% of pH 4 HTR could be achieved by VPZ 20 mg once daily and that 100.0% of pH 4 HTR and 6.8 of the mean 24 h intragastric pH could be attained by VPZ 20 mg twice daily, but not EPZ (Figure 3). Moreover, the acid inhibitory action of VPZ was not influenced by CYP2C19 polymorphism, meaning that the gastric acid inhibition required for eradication of the *H. pylori* by the dual therapy with AMOX can be achieved by VPZ 20 mg twice a day irrespective of CYP2C19 polymorphism.

## 3. Antibacterial Effect and Optimal Dosing Scheme of Amoxicillin

**Figure 4:**
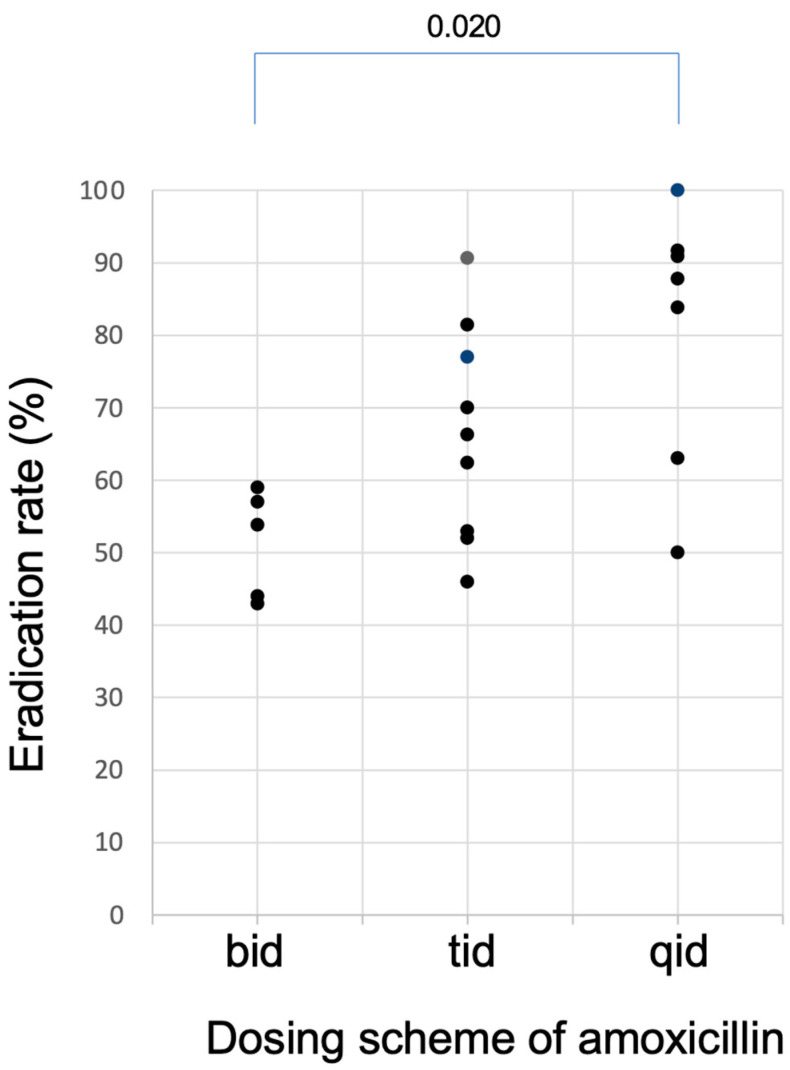
Plots of the eradication rates of studies of dual therapy with PPI and amoxicillin listed in Table 1 as a function of dosing schemes of amoxicillin. Eradication rates higher than 90.0% can be attained when amoxicillin was dosed at least three time daily (tid).

The relationship between the eradication rates and dosing scheme of AMOX is shown in Figure 4. When AMOX was dosed twice daily, the eradication rates were all less than 60%. To attain eradication rates higher than 90.0%, at least 3 times daily dosing seems necessary for AMOX in the dual therapy. Because the antibacterial effect of AMOX is time-dependent [33] and because it has no post antibiotic effect [34], its antibacterial activity depends on the percent time above MIC (%T > MIC) [35]. Since the plasma half-life of AMOX is as short as around 1 h, it will decrease below MIC in a few hours after dosing, and therefore, it is necessary to be dosed 3–4 times a day for AMOX to be effective. This is the reason why results of the twice-daily dosing regimens of AMOX are insufficient. In contrast, in reports that achieved high eradication rates, AMOX was administered 3–4 times daily when PPI was also administered at high doses 3–4 times a day.

There was no significant correlation between the total daily dose of AMOX and eradication rates in the regimens listed in Table 1 (*p* = 0.414)

Accordingly, to attain the sufficient eradication rates by the dual therapy with AMOX and an acid inhibitor, it is considered necessary to administer AMOX in 3 or more divided doses under the potent acid inhibition that can be attained by the higher doses of PPI dosed 3 or 4 times daily or VPZ 20 mg twice daily. Because the acid inhibitory effect of PPI is influenced by CYP2C19 polymorphism, but not for VPZ, VPZ is ideal and should be used for the dual therapy.

**Figure 5:**
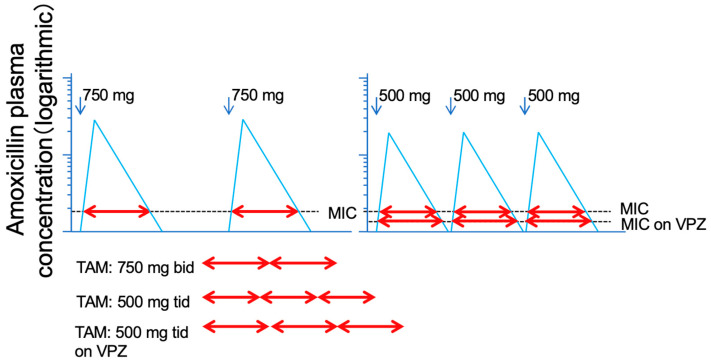
Logarithmic model of Time above MIC (T > MIC) of plasma level of amoxicillin 750 mg dosed twice and 500 mg three times daily. The total of T > MIC of amoxicillin 500 mg three times daily is longer than that of 750 mg twice daily. By using the vonoprazan, MIC is expected to be lowered, resulting in the further elongation of T > MIC of amoxicillin.

It is obvious that the time above the MIC obtained with 3 doses of AMOX 500 mg is longer than the time above the MIC obtained with 2 doses of AMOX 750 mg (Figure 5). Furthermore, since the MIC is lowered by strongly suppressing gastric acid, it is speculated that the time above the MIC will be even longer when an ultra-potent acid inhibitor, such as VPZ, is used.

## 4. Eradication Rates of Dual Therapy with Vonoprazan and Amoxicillin

The eradication rate reported in the first study of the dual therapy with VPZ and AMOX was 92.9%, which was identical to that of the triple therapy with VPZ, AMOX, and CAM [36]. In this report, there were no statistically significant differences in adverse events between dual therapy and triple therapy, but tended to be less with dual therapy. Therefore, it was considered that the dual VPZ/AMOX therapy could be a regimen for the first-line eradication therapy.

Since this report, there have been several studies on the dual therapy with VPZ and AMOX as listed in Table 2 [37,38,39,40,41,42,43,44,45]. Although the sufficient eradication rate could be attained by the first study [36], the eradication rates of the dual VPZ/AMOX therapies varies by different dosing schemes of AMOX and treatment periods as in the cases of PPIs.

As the duration of the treatment period, Lin et al. [42] reported that the dual VPZ/AMOX therapies for 7 days could not achieve an acceptable eradication rates (58.3% and 60.7%). Hu et al. [45] compared the 7 day and 10 day regimen with different AMOX dosing schemes and found that none of the 7 or 10 day regimens could attain the sufficient eradication rates. However, they reported that a 14 day regimen could attained a sufficient eradication rate (89.1%) [37]. However, studies by Suzuki et al. [38] Gotoda et al. [39] and Sue et al. [40], which were all from Japan, demonstrated that 7 days seemed a sufficient treatment period for the dual VPZ/AMMOX therapy. The optimal treatment periods for the dual VPZ/AMOX therapy seem to differ among different regions and ethnic groups.

Qian et al. [41] compared two types of ten-day dual VPZ/AMOX therapies with bismuth-containing quadruple therapy. The dual therapy with VPZ 20 mg bid and AMOX 750 mg qid attained the 93.4% of eradication rate, which was as high as that of the quadruple therapy (90.9%). Interestingly, the incidence of side effects of the dual therapy was significantly lower than that of the quadruple therapy. Zuberi [44] also reported that dual VPZ/AMOX therapy provides an acceptable and higher eradication rate (93.5%) with fewer adverse events in comparison with the triple therapy with omeprazole, CAM, and AMOX. Gao [43] reported that the dual VPZ/AMOX therapies were useful as the rescue therapy. However, the eradication rate of the report by Chey [46] was 78.5%, although the regimen was almost the same as that of study of Gao [43]. Accordingly, the optimal dosing schemes of the dual VPZ/AMOX therapy remains to be determined. The best regimens of the dual VPZ/AMOX therapy should be developed for each region and ethnicity.

## 5. Merits of the Dual Therapy with Vonoprazan and Amoxicillin

One of merits of the dual VPZ/AMOX therapy is to reduce the incidence of adverse events. As noted above, the incidence of adverse events observed in the dual therapies was lower in comparison with the triple therapy and quadruple therapy [36,41].

Pharmacologically, the greatest advantage of the dual therapy is that fewer drugs are used, reducing the risk of drug–drug interactions. CAM, which is often used in triple therapy, is a potent inhibitor of CYP3A4 and p-glycoprotein [47]. CAM has been reported to increase plasma levels of drugs metabolized and transported by these enzymes [29]. However, CAM is not involved in the dual VPZ/AMOX therapy. Therefore, the risk of drug–drug interactions related to CAM can be avoided. Of course, AMOX and VPZ are not without risk of interaction. VPZ has also been reported to affect the antiplatelet effects of Clopidogrel and Prasugrel [48], and has also been reported to affect CYPs [49]. Therefore, it cannot be said that the dual VPZ/AMOX is completely safe. However, it is considered safer than triple therapy from the viewpoint of drug–drug interactions. CAM also has an arrhythmia risk [50]. Therefore, dual therapy with VPZ and AMOX is particularly useful in patients with a risk of arrythmia, such as QT prolongation.

## 6. Dual Therapy with Vonoprazan and Amoxicillin with Reference to Clarithromycin Susceptibility

Suzuki et al. investigated influence of the CAM resistance in the comparative study of the dual therapy with VPZ 20 mg bid + AMOX 750 mg bid for 1 week and the triple therapy with VPZ 20 mg bid + CAM 20 mg bid + AMOX 750 mg bid for 1 week. In their study in patients infected with CAM-sensitive strains of *H. pylori*, the eradication rates of the dual and triple therapies were 85.5% and 95.1%, respectively. However, in patients infected with CAM-resistant strains of *H. pylori*, the respective eradication rates were 92.3% and 76.2% (*p* < 0.05). In other words, CAM has a negative effect on patients infected with CAM-resistant strains of *H. pylori*. There are many possible reasons, but one possible explanation is the pharmacodynamic antagonism between CAM and APMC.

In other bacteria, the combination of AMOX and CAM has been reported to be antagonistic [51,52]. The target of AMOX is PBP as noted above, which is the enzyme involved in biosynthesis of bacterial cell wall. When *H. pylori* grows, the expression of PBP is enhanced, which means that the expression of target of AMOX is increased, resulting in the bactericidal effect of AMOX being enhanced. In contrast, when the growth of *H. pylori* is inhibited, the expression of PBP is decreased. In this situation, the bactericidal effect of AMOX is decreased because the expression of target of AMOX is decreased. CAM is known as the inhibitor of rRNA, indicating that CAM inhibits the protein synthesis including PBP. Then, the sensitivity to AMOX is decreased by CAM.

However, if the patient is infected with CAM-sensitive strains, there is no problem because they can be killed by CAM. However, in cases infected with CAM-resistant strains of *H. pylori,* there might be some problems. MIC ranges of CAM-resistant strains of *H. pylori* are very wide (e.g., from 1 µg/mL to >32 µg/mL). In cases infected with CAM-weakly resistant strains of *H. pylori*, CAM will work halfway. Although *H. pylori* strains cannot be killed by CAM, the inhibitory effect of CAM on rRNA may affect the subsequent protein synthesis including PBP, leading to the reduced sensitivity to AMOX [53]. This seems to be the reason why the eradication rates attained by a PPI, AMOX, and CAM were reportedly very low in patients infected with CAM-resistant strains of *H. pylori* [54,55], although AMOX-resistant strains of *H. pylori* are rare.

## 7. Strategy for Patients Allergic to Penicillin

The dual therapy with VPZ and AMOX cannot be used for patients allergic to penicillin. Gao et al. [56] performed dual therapy with VPZ 20 mg bid and tetracycline 500 mg tid (body weight < 70 kg) or 500 mg qid (body weight ≥ 70 kg) for 14 days in patients who were allergic to penicillin or who had failed before. The eradication rate was 93.5%. Therefore, it is suggested that if one antibacterial agent with high susceptibility is selected besides AMOX, *H. pylori* eradication can be achieved with dual therapy with VPZ plus single effective antibacterial agent.

## 8. Conclusions

As mentioned above, the greatest merit of the dual VPZ/AMOX therapy is to reduce the risk of adverse events without lowering the eradication rates. In this regimen, CAM is not used, indicating the risk reduction of drug–drug interactions, arrhythmia, and other adverse events caused by CAM. The regimen is simple. There is also a report of the effective dual therapy with VPZ and single antimicrobial agent other than AMOX, such as tetracycline.

Now that we have vonoprazan in our hands, it is expected that eradication therapy will be reconstructed into a simpler regimen, such as dual therapy with VPZ and one antibacterial agent with high susceptibility (i.e., AMOX) in the optimal dosing scheme. It is also expected that we can break away from the stereotype of using multiple antibiotics to eradicate *H. pylori*.

Unfortunately, the use of VPZ is not widespread worldwide. It is hoped that VPZ will become widely available around the world in the future, and new drugs with similar effects will be developed, leading to the simplification of eradication regimens in many countries.

## Figures and Tables

**Table 1:** Lists of reports of dual therapy with PPI and AMOX.

Author	Year	Dosing Scheme of AMOX	Dosing Scheme of PPI	Duration	n	Eradication Rate (%) (ITT)
Furuta [8]	2001	500 mg qid	RPZ 10 mg qid	14 days	17	100.0%
Tai [9]	2019	750 mg qid	EPZ 40 mg tid	14 days	120	91.7%
Shirai [10]	2007	500 mg qid	RPZ 10 mg qid	14 days	66	90.9%
Bayerdorffer [11]	1995	750 mg tid	OPZ 40 mg tid	14 days	139	90.6%
Furuta [12]	2010	500 mg qid	RPZ 10 mg qid	14 days	49	87.8%
Miehlke [13]	2003	750 mg qid	OPZ 40 mg qid	14 days	38	83.8%
Furuta [5]	2001	500 mg tid	RPZ 10 mg bid	14 days	97	81.4%
Schwartz [14]	1998	1000 mg tid	LPZ 30 mg tid	14 days	51	77%
Miehlke [15]	2006	1000 mg tid	OPZ 40 mg tid	14 days	72	70%
Miyoshi [16]	2001	500 mg tid	OPZ 20 mg bid	14 days	98	66.3%
Nishizawa [17]	2012	500 mg qid	RPZ 10 mg qid	14 days	46	63.0%
Moiyoshi [16]	2001	500 mg tid	RPZ 10 mg tid	14 days	101	62.4%
Isomoto [18]	2003	1000 mg bid	RPZ 20 mg bid	14 days	63	59%
Wong [19]	2000	1000 mg bid	LPZ 30 mg bid	14 days	75	57%
Attumi [20]	2014	1000 mg bid	Dexlansoprazole 120 mg bid	14 days	13	53.8%
Schwartz [14]	1998	1000 mg tid	LPZ 30 mg bid	14 days	49	53%
Koizumi [21]	1998	500 mg tid	OPZ 20 mg qd	14 days	25	52%
Furuta [22]	1998	500 mg qid	OPZ 20 mg qd	14 days	62	50%
Bell [23]	1995	500 mg tid	OPZ 40 mg qd	14 days	60	46%
Cottrill [24]	1997	1000 mg bid	OPZ 40 mg qd	14 days	85	44%
Kagaya [25]	2000	750 mg bid	LPZ 30 mg qd	14 days	24	43%

Abbreviations: PPI = proton pump inhibitor, EPZ = esomeprazole, LPZ = lansoprazole, OPZ = omeprazole, RPZ = rabeprazole, qd = once daily, bid = twice daily, tid = three times daily qid = four times daily.

**Table 2:** Lists of reports of dual therapy with VPZ and AMOX.

Author	Year	Dosing Scheme of AMOX	Dosing Scheme of VPZ	Duration	n	Eradication Rate (%) (ITT)
Furuta [36]	2019	500 mg tid	20 mg bid	7 days	56	92.9%
Suzuki [38]	2020	750 mg bid	20 mg bid	7 days	168	84.5%
Gotoda [39]	2020	750 mg bid	20 mg bid	7 days	60	85.0%
Sue [40]	2022	500 mg qid	20 mg bid	7 days	20	90.0%
Lin [42]	2022	750 mg qid	20 mg bid	7 days	84	58.3%
Lin [42]	2022	500 mg qid	20 mg bid	7 days	61	60.7%
Hu [45]	2022	1000 mg bid	20 mg bid	7 days	24	66.7%
Hu [45]	2022	1000 mg bid	20 mg bid	10 days	37	89.2%
Hu [45]	2022	1000 mg tid	20 mg bid	7 days	21	81.0%
Hu [45]	2022	1000 mg tid	20 mg bid	10 days	37	81.1%
Qian [41]	2022	750 mg qid	20 mg bid	10 days	125	93.4%
Qian [41]	2022	1000 mg bid	20 mg bid	10 days	125	85.1%
Hu [37]	2022	1000 mg bid	20 mg bid	14 days	55	89.1%
Hu [37]	2022	1000 mg tid	20 mg bid	14 days	55	87.3%
Zuberi [44]	2022	1000 mg bid	20 mg bid	14 days	92	93.5%
Chey [46]	2022	1000 mg tid	20 mg bid	14 days	324	78.5%
Gao [43]	2022	1000 mg tid/750 mg qid	10 mg bid	14 days	43	95.3%
Gao [43]	2022	1000 mg tid/750 mg qid	20 mg bid	14 days	143	91.6%

Abbreviations: VPZ = vonoprazan AMOX = amoxicillin, bid = twice daily, tid = three times daily, qid = 4 times daily.

## Data Availability

We have used the published data.

## Abbreviations

AMOX = amoxicillin, CAM = clarithromycin, CYP = cytochrome P450, EPZ = esomeprazole, RPZ = rabeprazole, T>MIC = time above MIC, VPZ = vonoprazan.
